# General practitioner preferences for telehealth consultations in Australia: a pilot survey and discrete choice experiment

**DOI:** 10.1017/S1463423624000136

**Published:** 2024-05-09

**Authors:** Keshia R. De Guzman, Anthony C. Smith, Centaine L. Snoswell

**Affiliations:** 1 Centre for Online Health, The University of Queensland, Brisbane, Australia; 2 Centre for Health Services Research, The University of Queensland, Brisbane, Australia; 3 School of Pharmacy, The University of Queensland, Brisbane, Australia; 4 Centre for Innovative Medical Technology, University of Southern Denmark, Odense, Denmark

**Keywords:** COVID-19, economics, general practice, preferences, primary care, telehealth

## Abstract

**Aim:**

To identify and quantify general practitioner (GP) preferences related to service attributes of clinical consultations, including telehealth consultations, in Australia.

**Background:**

GPs have been increasingly using telehealth to deliver patient care since the onset of the 2019 coronavirus disease (COVID-19) pandemic. GP preferences for telehealth service models will play an important role in the uptake and sustainability of telehealth services post-pandemic.

**Methods:**

An online survey was used to ask GPs general telehealth questions and have them complete a discrete choice experiment (DCE). The DCE elicited GP preferences for various service attributes of telehealth (telephone and videoconference) consultations. The DCE investigated five service attributes, including consultation mode, consultation purpose, consultation length, quality of care and rapport, and patient co-payment. Participants were presented with eight choice sets, each containing three options to choose from. Descriptive statistics was used, and mixed logit models were used to estimate and analyse the DCE data.

**Findings:**

A total of 60 GPs fully completed the survey. Previous telehealth experiences impacted direct preferences towards telehealth consultations across clinical presentations, although in-person modes were generally favoured (in approximately 70% of all scenarios). The DCE results lacked statistical significance which demonstrated undiscernible differences between GP preferences for some service attributes. However, it was found that GPs prefer to provide a consultation with good quality care and rapport (*P* < 002). GPs would also prefer to provide care to their patients rather than decline a consultation due to consultation mode, length or purpose (*P* < 0.0001). Based on the findings, GPs value the ability to provide high-quality care and develop rapport during a clinical consultation. This highlights the importance of recognising value-based care for future policy reforms, to ensure continued adoption and sustainability of GP telehealth services in Australia.

## Introduction

General practitioners (GPs) have been increasingly using telehealth to deliver patient care since the onset of the 2019 coronavirus disease (COVID-19) pandemic (Breton *et al.*, 2021, Goodyear-Smith, 2021, Ray and Mash, 2021, Smith *et al.*, 2020). In Australia, the delivery of telehealth services in general practice has been supported by increased telehealth funding opportunities, introduced by the Commonwealth Government in March 2020 (Jonnagaddala *et al.*, 2021, De Guzman *et al.*, 2022b). Prior to the pandemic, telehealth uptake by GPs in Australia was very low because of the absence of government funding for GP telehealth services. The introduction of increased telehealth funding included remuneration for both telephone and videoconference consultations, provided by GPs directly to their patients through a fee-for-service system (Snoswell *et al.*, 2020). While the funding scheme supports both telephone and videoconference consultations, the majority (around 97%) of telehealth consultations provided by Australian GPs to date have been done by telephone (De Guzman *et al.*, 2022b, Snoswell *et al.*, 2020). Similar trends have been observed globally, with telephone being the main choice of telehealth modality. In a study reported in England during 2021, videoconference consultations accounted for less than 0.5% of GP consultations (Greenhalgh *et al.*, 2022). In Canada, a very small number of primary care providers (around 4%) reported frequent use of videoconferencing for patient consultations (Breton *et al.*, 2021).

While telephone consultations certainly have a role in general practice for short interactions (such as follow-up conversations or provision of test results) (Mckinstry *et al.*, 2009), evidence is emerging which demonstrates that videoconference consultations may be more effective than telephone consultations. The visual component of videoconference consultations has been shown to increase diagnostic accuracy, improve patient to clinician rapport and potentially enable higher quality of care (Snoswell *et al.*, 2021a, Rush *et al.*, 2018, Donaghy *et al.*, 2019). However, GPs have consistently used telephone, which has led to concerns that telehealth services cannot achieve high-quality care (Greenhalgh *et al.*, 2022, De Guzman *et al.*, 2022a, Scott *et al.*, 2021). Some GPs have also been reluctant to embrace telehealth models of care, even though there are many advantages for providing these services (eg, greater convenience and less burden for the patient, timely access to care) (Scott *et al.*, 2021). This means that reasons which influence GP willingness to use telehealth are important in understanding how to encourage best integration of telehealth service models into usual care.

Existing research has explored some of the driving forces behind high GP telephone use in Australia. (Jonnagaddala *et al.*, 2021, White *et al.*, 2022, De Guzman *et al.*, 2021a). These include time pressures in general practice settings, the convenience and ease of using the telephone to connect with patients, and poor GP experiences with videoconferencing (Jonnagaddala *et al.*, 2021, White *et al.*, 2022, De Guzman *et al.*, 2021a). Internationally, studies have found that multiple factors impact GP telehealth delivery, highlighting the complexity of implementing GP telehealth services into general practice (De Vera *et al.*, 2022, Reeves *et al.*, 2021). A review article outlined how the consultation type, patient characteristics and patient presentation are integral to determining the appropriateness of telehealth use (Reeves *et al.*, 2021). Some studies have reported GPs preferences for telephone consultations; however, very little is known in the context of the Australian primary care setting. GP preferences for alternative service models, such as telehealth, play an important role in the uptake and sustainability of these services. Methods to assess clinician preferences for healthcare services have been taken from other disciplines, such as economics, and are becoming more commonly used (Ryan, 2004, Clark *et al.*, 2014). Using a discrete choice experiment (DCE) method, GP preferences for different service attributes of clinical consultations can be compared. The aim of this pilot study was to identify GP preferences for service attributes of clinical consultations in Australia and to quantify the value of these attributes. This information may be useful for the development of strategies that encourage videoconference use, both in Australia and in other settings.

## Method

A survey was used to ask GPs general questions and have them complete a DCE. The general questions explored GP telehealth experiences, while the DCE elicited and quantified GP preferences for various service attributes of clinical (telehealth and in-person) consultations. Ethics approval was received from The University of Queensland Human Research Ethics Committee (2022/HE000213).

### General questions

The general questions included (1) *demographic questions,* (2) *telehealth questions* and (3) *direct preference questions*. Demographic questions covered sociodemographic characteristics of the GP participants which included age, gender, education, practice experience, practice setting and practice location. Additional questions related to practice ownership, primary billing source and average hourly income before tax. Telehealth questions covered topics related to previous telehealth knowledge or experience, resources required for different consultation modes and the impact of bulk-billing restrictions on GP practice. Direct preference questions asked GPs to indicate which mode they preferred for varying consultation lengths and different patient clinical presentations.

### DCE method description

A DCE is a quantitative method that is increasingly being used in health-related research (Ryan, 2004, Clark *et al.*, 2014, Reed Johnson *et al.*, 2013). In a DCE, participants are presented with alternative hypothetical scenarios, known as choice sets, which contain different levels of predefined service attributes (Ryan, 2004, Reed Johnson *et al.*, 2013). In this case, the DCE scenarios contained different levels for service attributes of consultations, including in-person and telehealth alternatives. Based on random utility theory, the DCE assumes that individuals make rational choices in order to maximise their utility; this enables the estimation of their preferences for service attributes, based on their response to the scenarios presented to them in each choice set (Manski, 2001, Bayoumi, 2004, Reed Johnson *et al.*, 2013). In this study, the GP participants were asked to make direct trade-offs to reveal their relative preferences and the strength of those preference for service attributes of clinical consultations (in-person, telephone and videoconference modes).

### DCE development and sample size

**Figure 1.**
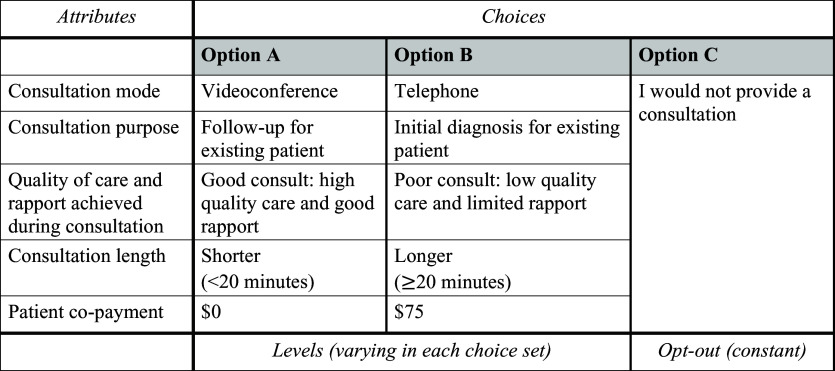
Choice set one indicating an example of the discrete choice experiment question

The DCE scenarios in the survey were developed based on qualitative work conducted by the research team (De Guzman *et al.*, 2021a), and review of the literature, which is an important part of DCE development (Vass *et al.*, 2017). The qualitative work identified factors that influence GP choice of consultation mode, including the ability to provide quality of care, the purpose of the consultation, and the presence of time and reimbursement considerations (De Guzman *et al.*, 2021a). These factors were identified from themes that came out of qualitative interviews with GPs, which helped to choose important service attributes of clinical consultations for investigation in this DCE study. Review of the literature also identified previous surveys that investigated GP telehealth use, which also informed the DCE questions (Chudner *et al.*, 2019, Manski-Nankervis *et al.*, 2022, Taylor *et al.*, 2021, Snoswell *et al.*, 2021b). Discussions between a team of experienced GPs, telehealth experts, and researchers were undertaken to confirm and refine the DCE service attributes so that the DCE survey could be finalised. Five service attributes (key variables) for telehealth consultations with varying levels were included in the DCE, which were consultation mode, consultation purpose, consultation length, quality of care and rapport, and patient co-payment. Each of these service attributes were assigned two to three levels that described different consultation characteristics that were possible for that attribute. The DCE tool was designed to be d-efficient to ensure that participants are provided with a reasonable number of choice sets, whilst still identifying meaningful attribute-level combinations (Ryan, 2004). Using this design, eight choice sets (eight DCE questions) were presented to the participants in the final survey. GPs were asked to select one of three options in each DCE choice set, which included an option between two consultation options with varying levels of service attributes, or an option to not provide the consultation (opt-out option) (Figure 1). To ensure that realistic choice sets were presented, GPs could choose ‘not to provide the consultation’. Additionally, consultation length was separated into less than, or greater than 20 min, which corresponds to GP consultation types in Australia. The patient co-payment represented the minimum monetary amount that the GP was ‘willing to accept’ to provide the consultation. The sample size for the DCE was calculated based on the Johnson and Orme rule of thumb formula, which recommended a minimum sample size of 62.5. The Johnson and Orme rule was applied as this is a recommended formula for calculated minimum sample sizes in DCE studies; it considers the number of choice sets, alternatives and levels (De Bekker-Grob *et al.*, 2015, Speckemeier *et al.*, 2021).

### GP recruitment and survey administration

GPs working in Australia were invited to complete the general survey and DCE online, which took approximately 10 minutes to complete all questions. Recruitment of GPs occurred through networking and snowball sampling, as well as via social media, university news channels, professional membership organisations, and private primary care organisations. The survey was administered using the Qualtrics online survey platform, and GP participants provided consent prior to online survey completion. GP participants who completed the survey were also given the option to go into a draw for one of three prizes: a $500 gift card, or one of two $100 gift cards.

### Data analysis

Sociodemographic details, general telehealth experience and direct preference questions were analysed using descriptive statistics and graphical representation. These statistics were separated and presented according to GP telehealth experiences: telephone and videoconference experience, or telephone experience only. The DCE questions were analysed using a mixed logit model with categorical attribute levels specified using dummy coding for all variables expect the continuous co-payment variable. The choice for ‘I would not provide a consultation’ and the ‘co-payment’ variable were specified to have a constant (fixed) effect within the model, and all other variables were considered random with normal distributions. To ensure that the model was robust, 500 Halton draws were run which indicates the number of unique times the mixed logit model is run. The model was investigated for main effects, so no interaction terms were included. The mixed logit model incorporates preference heterogeneity in the sample, as the coefficients are treated as random, which was examined through the relative magnitude of the standard deviation compared to the coefficient. The model was run with the entire sample and then separated according to GP telehealth experiences (videoconference and telephone, or telephone only) as well. Standard errors and 95% confidence intervals were used to estimate uncertainty of the results, with *P*-values of <0.05 used as measures for statistical significance. All analyses were undertaken in Microsoft Excel® (v365) and Stata® (v16.0) statistical software.

## Results

There were 60 GP participants that fully completed the survey, which included both the general questions and DCE questions. As the survey was sent through multiple channels and advertised through snowballing, it was not possible to calculate a completion (%) rate as the total number of invited individuals was unknown.

### GP sociodemographic characteristics

Most GP participants were male (52%) and aged between 31 and 40 years (50%) (Table 1). Half of the participants (50%) had received an undergraduate degree as their highest level of training, with some who had completed a graduate certificate or diploma (22%), or a professional or research masters (22%). The length of time that GP participants had been registered ranged widely (from 1 to over 15 years), with most (37%) registered for one to five years. The participants represented general practice care in both metropolitan areas (57%), or regional, rural and remote settings (43%). Within work history, almost half of the GPs (45%) worked in metropolitan settings only, or mainly metropolitan settings, with some having experience in rural and remote areas (40%). Over half of the GPs indicated that Queensland was their primary state of practice (57%), although GPs also worked across Victoria (20%), New South Wales (10%), South Australia (7%), Australian Capital Territory (5%) and The Northern Territory (2%). Varying levels of practice ownership were reflected, with half of the GPs being sole owners (50%), and others being part owners (22%) or non-practice owners (28%). Most GPs charged an out-of-pocket cost for non-concessional patients (75%), and the primary billing source was relatively balanced across mixed billing (52%) and exclusively bulk billing (48%).

**Table 1.** Sociodemographic characteristics of GP participants (*n* = 60)

Characteristic		*n* (%)
Age	21–30 years	13 (21.7)
	31–40 years	30 (50.0)
	41–50 years	6 (10.0)
	51–60 years	10 (16.7)
	Over 60 years	1 (1.7)
Gender	Male	31 (51.7)
	Female	26 (43.3)
	Other	3 (5.0)
Highest level of training	Undergraduate or undergraduate (hons)	30 (50.0)
	Graduate certificate or diploma	13 (21.7)
	Professional Masters	6 (10.0)
	Master of Philosophy (MPhil)	7 (11.7)
	Doctor of Philosophy (PhD)	4 (6.7)
Time as registered GP	Less than 1 year	13 (21.7)
	1–5 years	22 (36.7)
	6–10 years	13 (21.7)
	11–15 years	4 (6.7)
	Over 15 years	8 (13.3)
Primary location	Metropolitan	34 (56.7)
	Regional	22 (36.7)
	Rural or remote	4 (6.7)
Work setting history	Metropolitan only	27 (45.0)
	Mainly metropolitan, some rural and remote	24 (40.0)
	Mainly rural and remote, some metropolitan	6 (10.0)
	Only rural and remote	3 (5.0)
Primary state of practice	Queensland	34 (56.7)
	New South Wales	6 (10.0)
	Victoria	12 (20.0)
	South Australia	4 (6.7)
	Western Australia	0 (0.0)
	The Northern Territory	1 (1.7)
	Australian Capital Territory	3 (5.0)
	Tasmania	0 (0.0)
Practice ownership	Yes – sole practice owner	30 (50.0)
	Yes – part practice owner	13 (21.7)
	No	17 (28.3)
Charge OOP (out of pocket costs) for non-concessional patients	Yes	45 (75.0)
	No	12 (20.0)
	Other	3 (5.0)
Primary billing source	Exclusively bulk billing	29 (48.3)
	Mixed billing	31 (51.7)
	Exclusively private billing	0 (0.0)
		Mean (SD)
Average hourly income ($) before tax		58. 5 (77.6)

### General telehealth questions

#### Videoconference and telephone experience

GP participants indicated that they were relatively experienced and knowledgeable with telehealth consultations (average score = 7.3 on scale from 1 to 10). However, of the 60 participants, 55% (n = 33) had never conducted a videoconference consultation with a patient, meaning that they only had telephone consultation experience. For the other 45% (n = 27) of participants who did have videoconference experience, they reported an average of six months (ranged from 3 to 24 months) experience delivering videoconference consultations. Most GPs (80%) felt that the bulk-billing restrictions had a positive effect or no effect on their GP businesses. About 20% of GPs thought that the bulk-billing restrictions had a negative impact on their practice (Supplementary file 1).

#### Resources required for videoconference consultations compared to in-person consultations (n = 27)

**Figure 2.**
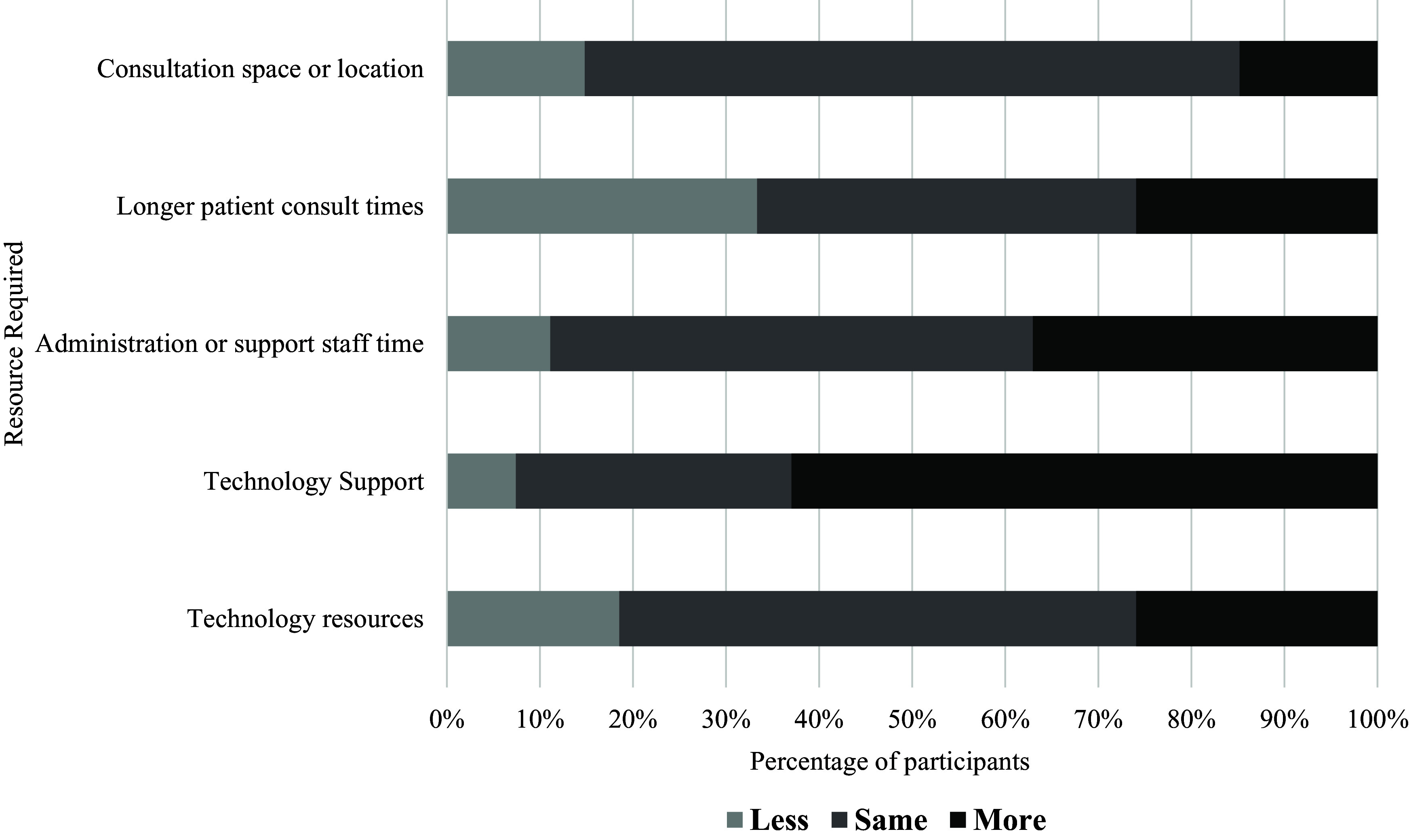
Resources required for videoconference consultations compared to in-person consultations for GPs with videoconference experience (*n* = 27)

Approximately half of the participants indicated that the same amount of technology resources (56%) and administration support (52%) would be needed to deliver videoconference consultations compared to in-person consultations (Figure 2). Most participants (63%) also felt that more technology support was required for videoconference than in-person modes. For consultation space, the majority of participants (70%) felt that this resource would be the same for videoconference consultations and in-person consultations. Most participants (41%) thought that videoconference consultations would require the same length of time as in-person consultations, although some (33%) thought it would require less time. Overall, few participants felt that videoconference consultations would require less resources compared to in-person consultations, and most indicated that the same amount, or more resources, would be required for videoconference delivery (Figure 2).

#### Resources required for telephone consultations compared to in-person consultations (n = 60)

**Figure 3.**
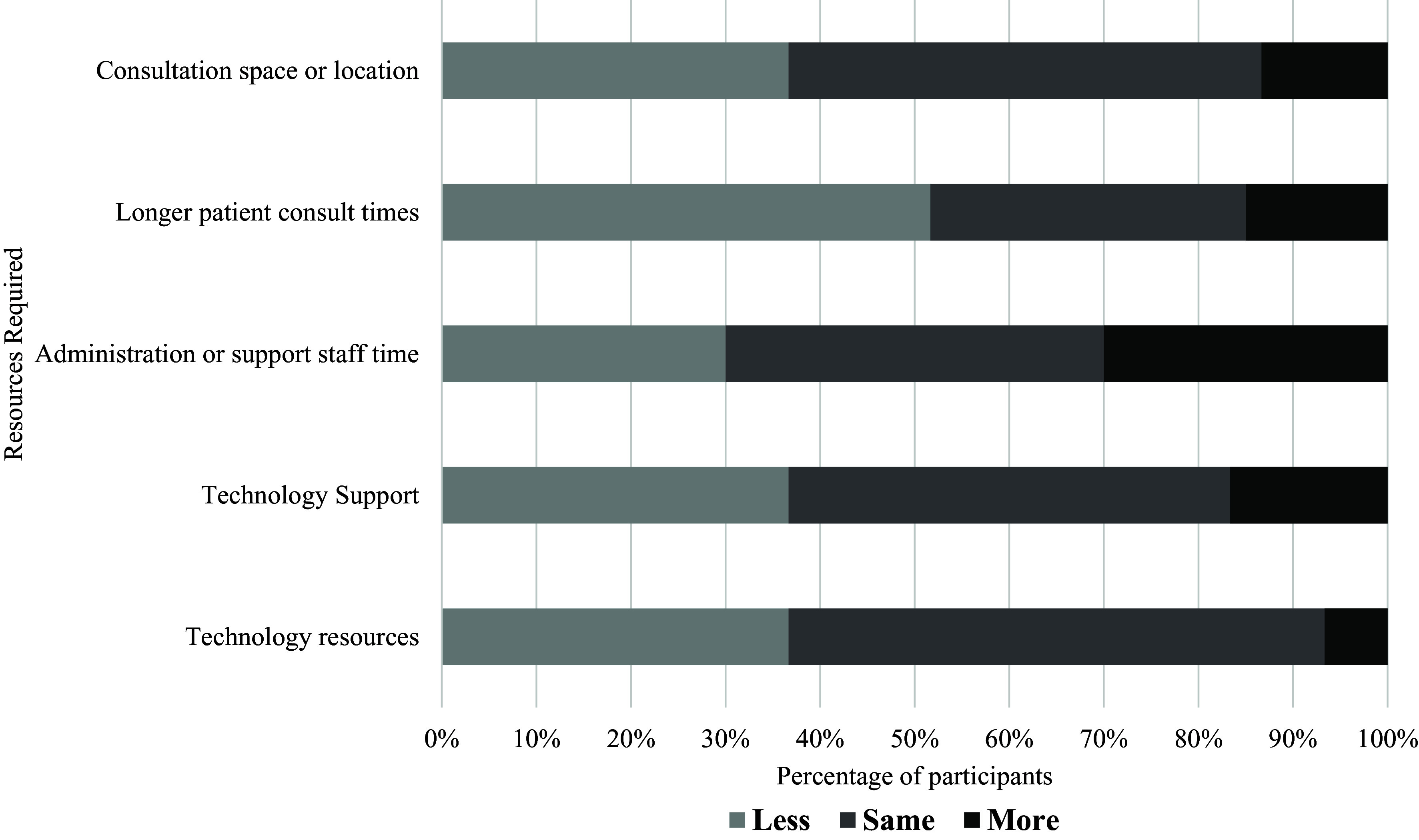
Resources required for telephone consultations compared to in-person consultations (*n* = 60, all respondents)

Approximately half of the participants felt that the amount of technology resources (57%), technology support (47%) and consultation space (50%) would be the same for telephone consultations compared to in-person consultations (Figure 3). Very few participants (7%) thought that more technology resources would be required to deliver telephone consultations. Most participants (40%) felt that the same amount of administration support would be required for telephone and in-person consultations; however, a balanced proportion of participants felt that either less (30%) or more (30%) administration support would be required. Unlike videoconference consultations, over half of the participants (52%) felt that delivering telephone consultations would require shorter lengths of time than in-person consultations. Overall, few participants felt that telephone consultations would require more resources than in-person consultations, with most indicating that the same amount, or less resources, would be required for telephone delivery.

### Direct GP preferences

#### Preferences for consultation mode for different consultation lengths

Overall, for a short 5-minute consultation, there was a preference for using the telephone (53%) to engage with patients, compared to an in-person appointment (20%) or videoconference consultation (10%). Some GPs (17%) expressed no preference of modality for a short consultation (Supplementary file 1). For a long 40-minute consultation, almost half of the GPs (43%) indicated a preference for an in-person consultation, compared to videoconference (33%) or telephone (17%) consultations. These preferences differed according to their previous telehealth experiences. Participants who had videoconference experience (n = 27) demonstrated much higher preference for telephone consultations (70%) for a 5-minute consultation, compared to those who only had telephone experience (39%). Participants with videoconference experience also demonstrated a much higher preference for videoconference consultations (41%) for a 40-minute consultation, compared to those who only had telephone experience (27%). Interestingly, none of the participants with videoconference experience preferred videoconference consultations for a 5-minute consultation, or telephone consultations for a 40-minute consultation.

#### Preferences for consultation modes for different patient presentations, telephone experience only (n = 33)

**Figure 4.**
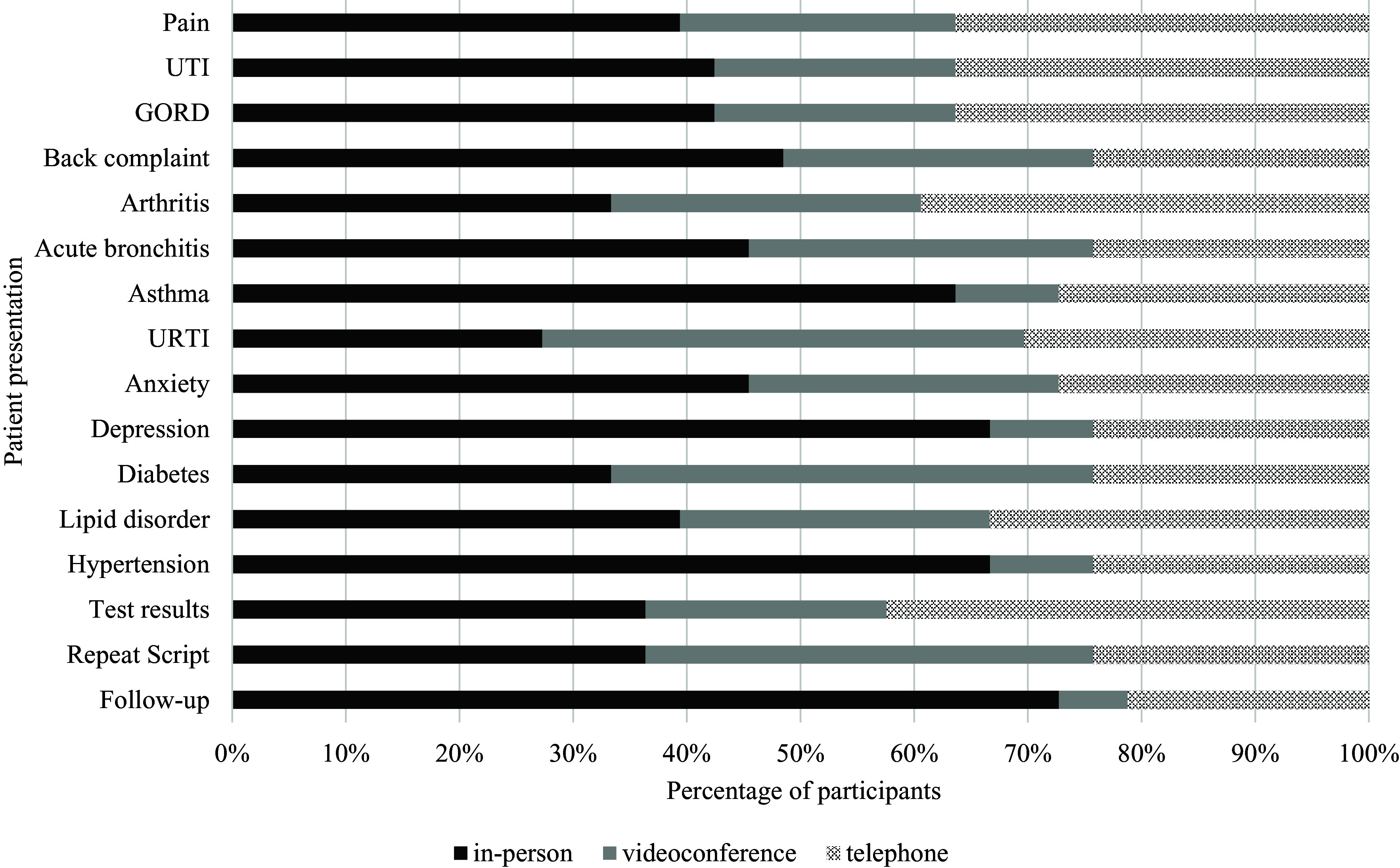
Preferred modality across various patient presentations for general practitioners with telephone experience only (*n* = 33)

The preferences for consultation modes for different presentations varied based on personal experience with telephone and videoconference consultations. For those with telephone experience only, in-person consultations were preferred across most patient presentations (69% of all cases), with the exception of repeat prescriptions, test results, diabetes reviews, upper respiratory tract infections (URTIs) and arthritis (Figure 4 and supplementary file 1). Videoconference consultations were only slightly preferred over in-person consultations for repeat prescriptions (39% versus 36%) and diabetes (42% and 33%) presentations. For URTIs, both telephone (30%) and videoconference (42%) consultations were preferred, over in-person consultations (27%). For test results and arthritis presentations, telephone consultations were preferred over videoconference and in-person consultations.

#### Preferences for consultation modes for different patient presentations, videoconference and telephone experience (n = 27)

**Figure 5.**
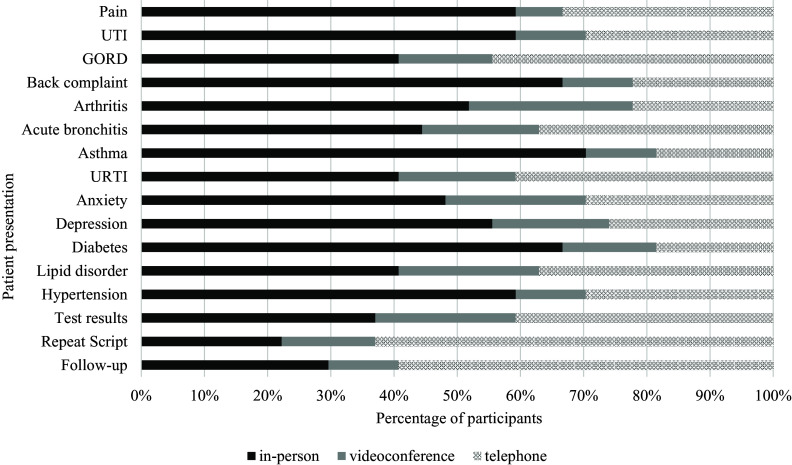
Preferred modality across various patient presentations for general practitioners with telephone and videoconference experience (*n* = 27)

For participants with both videoconference and telephone experience, in-person consultations were preferred across most patient presentations as well (69% of all cases) (Figure 5 and supplementary file 1). For the presentations where in-person was not preferred, telephone consultations were preferred in situations that included patient follow-up (59%), repeat prescriptions (63%), test results (41%), URTIs (41%) and gastro-oesophageal reflux disease (44%). The proportion of participants who would prefer telephone consultations ranged from 19% to 63% across different patient presentations. This was higher than the proportion of participants who would prefer videoconference consultations, which ranged from 8% to 26% across patient presentations. Interestingly, for participants who had telephone experience only, preferences for videoconference consultations were sometimes higher for different patient presentations (6%–42%) and lower for telephone consultations across different patient presentations (24%–43%). Irrespective of participant experiences with telephone or videoconference consultations, in-person appointments were the primary preferred method for most patient presentations.

### GP preferences from DCE

**Figure 6.**
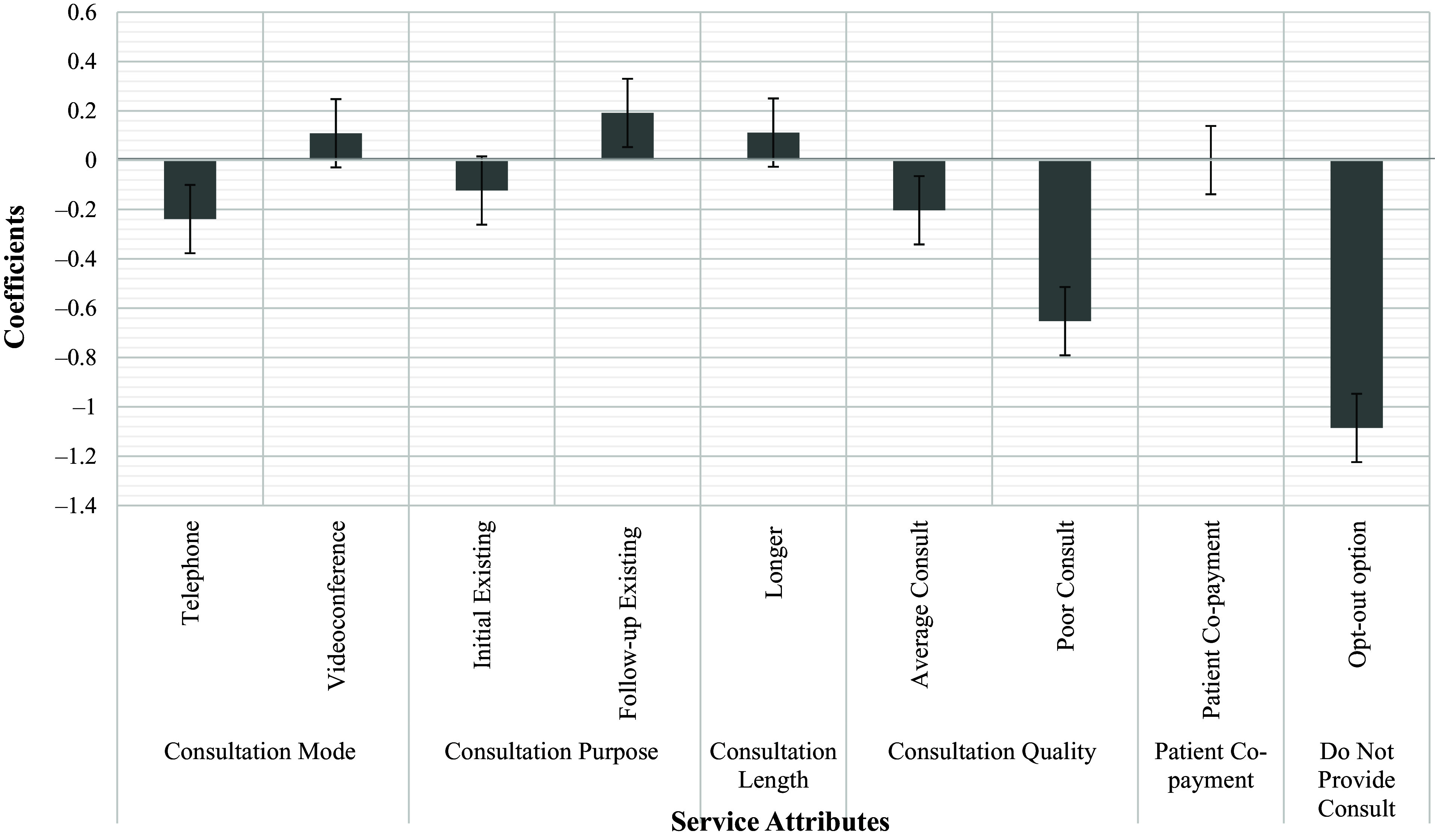
Preference coefficients for the mixed logit model. *Reference levels: in-person (consultation mode), initial new (consultation purpose), shorter (consultation n length), good consult (consultation quality)

Across all responses, GPs selected to ‘not provide the consultation’ across approximately 17% of the eight scenarios. Most of the model coefficients were small and statistically insignificant, demonstrating undiscernible differences between GP preferences for some of the service attributes (Table 2). Consistent with expectations, GPs seemed to have a slight preference towards longer consultations than shorter consultations, follow-up consultations for existing patients over initial diagnosis for new patients and in-person to telephone consultations. The model coefficients for consultation modes, of telephone and videoconference, were nearly zero, which may indicate that there is potentially a neutral preference for telehealth consultations compared to in-person consultations. Similar to consultation mode, the coefficients for different consultation purposes (initial diagnoses or follow-up presentations) were also nearly zero, demonstrating that GPs may have no prominent preference towards consultation purpose. GP preference for patient co-payment was slightly above zero, indicating a very small positive preference for higher payment consultations, although this was statistically insignificant (*P* = 0.862). Unsurprisingly, GPs preferred a good consult, which meant a consultation with high-quality care and good rapport, to an average or poor consult. GPs preferred to have a high-quality consult with good rapport, over a lower quality consult with limited rapport, which was statistically significant (*P* = 0.002). GPs also preferred to provide a consultation, rather than not provide a consultation, demonstrating that GPs prefer to provide care to their patients than decline a consult based on the attributes included in the scenarios (*P* < 0.0001). The model coefficients were plotted to visually illustrate the comparative preferences for GPs towards different service attributes (Figure 6). It can be seen that GPs likely value consultation quality over other service attributes, as a good consult was more greatly preferred over a poor consult; the coefficients for quality of care were also larger than those for the other service attributes. The coefficients for consultation mode, consultation purpose and consultation length were closer to zero, highlighting that GPs may have neutral preferences towards these service attributes. GPs prefer to provide a consultation to their patients, which may indicate that they would provide care, irrespective of the consultation mode, length or purpose (Figure 6). The DCE analysis was also run according to GP telehealth experiences (videoconference experience and no videoconference experience), but no differences in the coefficients was evident.

**Table 2.** Mix logistical regression model outputs from the discrete choice experiment

Service attribute	Attribute levels	Coefficient	Standard error	*P*-value	95% CI	SD for distribution	
						SD	*P*-value
Consultation mode (type)	In-person	Reference					
	Telephone	−0.2387	0.1985	0.229	−0.6277 to 0.1504	0.7336	0.007
	Videoconference	0.1091	0.1769	0.537	−0.2376 to 0.4558	0.6851	0.009
Consultation purpose	Initial diagnosis for new patient	Reference					
	Initial diagnosis for existing patient	−0.1231	0.1753	0.483	−0.4668 to 0.2206	0.4131	0.231
	Follow-up for existing patient	0.1917	0.1982	0.334	−0.1968 to 0.5801	0.7489	0.003
Consultation length	Shorter (<20 min)	Reference					
	Longer (>20 min)	0.1119	0.1423	0.432	−0.1670 to 0.3907	0.0021	0.994
Quality of care and rapport	Good consult: high-quality care and good rapport	Reference					
	Average consult: high-quality care and some rapport	−0.2030	0.1845	0.271	−0.5645 to 0.1586	0.2455	0.617
	Poor consult: Low-quality care and limited rapport	−0.6525	0.2095	0.002	−1.0631 to –0.2418	0.9347	<0.001
Patient co-payment	Cost charged to the patient each time a consult is delivered	0.0003	0.0016	0.862	−0.0028 to 0.0034		
Do not provide the consultation	Not applicable, described in survey pre-amble	−1.0855	0.2091	<0.0001	−1.4954 to –0.6756		

CI = confidence interval, SD = standard deviation.

Model fit characterised by an Akaike information criterion of 985 and a Bayesian information criterion of 1019.

## Discussion

This study identified and quantified GP preferences for service attributes of clinical consultations, including telehealth consultations, in Australia. Compared to in-person consultations, participants felt that telephone would require the same or less resources, while videoconference typically demanded more resources. Telephone was the preferred modality for short 5-minute consultations, while videoconference or in-person modes were preferred for longer 40-minute consultations. Across patient presentations, in-person consultations were still preferred (in approximately 70% of all scenarios). The DCE results showed undiscernible differences between GP preferences of some service attributes, including consultation purpose, duration or mode (in-person or telehealth), as the coefficients for these attributes were not statistically significant. However, providing a good consultation with high-quality care and good rapport was preferred by GPs, demonstrating that GPs value the ability to provide high-quality care to their patients. Overall, the results show that GPs would prefer to provide care to their patients rather than decline care on the basis of consultation purpose, length or mode.

Over half of the GP participants reported that they had never provided a videoconference consultation, reflecting greater telephone experiences in Australia (De Guzman *et al.*, 2022b). Overall, telephone was preferred for a 5-minute consultation, which is consistent with research that supports telephone consultations for single-issue concerns (Reeves *et al.*, 2021, Imlach *et al.*, 2020, Hewitt *et al.*, 2010, Carrillo De Albornoz *et al.*, 2022, Snoswell *et al.*, 2022). GPs with both telephone and videoconference experience had a higher preference for choosing telephone for follow-up, repeat prescription and test result presentations, which is observed in existing literature (Hewitt *et al.*, 2010, Carrillo De Albornoz *et al.*, 2022, Reeves *et al.*, 2021). Interestingly, GPs with videoconferencing experience predominantly chose telephone for 5-minute consultations and exclusively chose videoconference or in-person modes for 40-minute consultations. However, GPs who did not have videoconferencing experience preferred to deliver 40-minute consultations by telephone or in-person modes. This highlights that GPs who are more experienced telehealth users (ie, have videoconference experience) may feel that the telephone is less appropriate for long consultations and complex issues. In Australia, reimbursement for telephone consultations over 20 minutes was removed from the national health fund in July 2021 (De Guzman *et al.*, 2022b, Woodley, 2021). While the funding decision aligns with evidence that supports short telephone consultations, this could increase the potential for over-servicing. There is also concern that the absence of funding for long telephone consultations creates access issues for patients without the capacity to receive videoconference care (eg, limited internet, no infrastructure, rural and remote patients) (Thomas-Jacques *et al.*, 2021, Woodley, 2021).

Interestingly, overall preference for videoconference consultations across different patient presentations was higher for those who only had telephone experience, compared to those with videoconferencing and telephone experience. This could be because GPs who have previously delivered videoconference consultations may have had poor experiences with videoconference delivery. A UK study reported that while advances in digital technologies and telehealth policies (eg, available funding) have been made, many GPs have issues with videoconferencing usability and functionality (Greenhalgh *et al.*, 2022). GPs often report that telephone is easier and more convenient to deliver than videoconference consultations (Rush *et al.*, 2018, Greenhalgh *et al.*, 2022, De Guzman *et al.*, 2022a). Similarly, this study highlighted the ease of delivering a telephone consultation, as less resources are required than an in-person or videoconference consultation. An Australian study found that telephone consultations could be provided with existing resources (Snoswell *et al.*, 2022), reporting that resource requirements (administration, technical support and hardware) for videoconference consultations were much higher. Another Australian survey found that the increase in GP telehealth uptake has mainly been a function of COVID-19 and the expansion of telehealth funding (Scott *et al.*, 2021). The recognition of telehealth as a key strategy to prevent widespread COVID-19 transmission (Breton *et al.*, 2021, Smith *et al.*, 2020) was reflected in this study as GPs preferred telehealth consultations for URTIs. As the impacts of COVID-19 settle and policies are reviewed, the benefits of telehealth use that exist outside of the pandemic need to be recognised, such as opportunities to expand the capacity of the primary care sector and achieve wider societal benefits (De Guzman *et al.*, 2021b).

The results from the DCE demonstrated that GPs would prefer to provide a patient consultation, rather than decline a patient consultation. This demonstrates that irrespective of consultation mode (in-person or telehealth), GPs would still be inclined to provide care to their patients. Many studies have confirmed that both patients and GPs highly value the ability to develop therapeutic rapport and that many GPs strive to improve the quality of care they provide to their patients (De Guzman *et al.*, 2021a, Donaghy *et al.*, 2019, Ahmed *et al.*, 2021, Grol *et al.*, 1985). This is consistent with the findings from this study which shows that GPs value the ability to provide a consultation with high-quality care and good rapport. When considering this, it is interesting that high GP telephone use has been observed, when there is evidence that videoconference consultations can achieve greater rapport building, visual interaction and in-depth discussion (Manski, 2001, Carrillo De Albornoz *et al.*, 2022, Yao *et al.*, 2022, Snoswell *et al.*, 2021a). Some explanations for this is that GPs have been found to have variable confidence with videoconference delivery, or some perceive the benefit of videoconference over telephone as minimal (Greenhalgh *et al.*, 2022). Innovations that are perceived to have minimal relative advantages, compared to existing services, are often quickly abandoned (Greenhalgh *et al.*, 2022, Safaeinili *et al.*, 2020). This supports the need for research that assesses and confirms the relative effectiveness of videoconference consultations, compared to telephone consultations. The generation of this supporting evidence will be paramount in achieving continued telehealth adoption post-pandemic and increasing videoconference use in primary care. In addition, a skilled workforce that has received telehealth education and training is essential (Thomas *et al.*, 2022). GPs who have not received telehealth training are usually less likely to change their practice towards telehealth use (Thomas *et al.*, 2022). Given that GPs highly value quality of care, consideration towards the incorporation of value-based care in future funding reforms will be important (De Guzman *et al.*, 2022a).

### Limitations

GPs were invited to participate in this study through a range of professional networks and channels. It is possible that GPs who opted to participate in this study had an interest in telehealth, resulting in potential bias. The sample size for this pilot study was small and was just under the calculated minimum sample size for this study using the Johnson and Orme rule of thumb. However, it is unlikely that increasing the sample size by a marginal amount would yield statistically significant results, as the population of GPs in Australia is very large (ie, approximately 30 972 full-time equivalent GPs in 2021 (Australian Government Department of Health and Aged Care, 2022). Based on this population size, and using a general sample size calculation with standard values (proportion, confidence levels and confidence intervals), 385 GPs would be needed (Australian Bureau of Statistics, 2022). This also means that the study sample may not be representative of the entire GP population in Australia and that the findings need to be confirmed in a larger sample of Australian GPs. Another limitation is that DCE responses from GPs may not align with their actual behaviour; in that, participants may say one thing and take different actions in practice. However, the DCE method does provide insights into preferences that are not revealed. Given the insignificant coefficients for most of the service attributes, willingness to accept amounts were not calculated.

## Conclusion

This study provides valuable insights into GP preferences for service attributes of clinical consultations and telehealth delivery in general practice settings. Based on the findings, GPs value the ability to provide high-quality care, and to develop rapport, during a clinical consultation. With emerging evidence in the literature supporting the additional benefits of videoconference consultations compared to telephone consultations, it is important to understand factors which influence choice of telehealth adoption in general practice. This study showed that GPs generally prefer to provide care to their patients, irrespective of the consultation mode, length or purpose. Prior GP experiences with telehealth services also impact preferences towards telehealth care for various clinical presentations. Overall, traditional in-person consultation modes are largely favoured, and telephone consultations are perceived as much easier to deliver than videoconference consultations. Future evidence which compares the effectiveness of videoconference consultations to telephone consultations is needed to influence GP willingness to practice telehealth in the primary care sector. The recognition that value-based care is important for future policy reforms will be needed to ensure the continued adoption and sustainability of GP telehealth services.

## Supporting information

De Guzman et al. supplementary material De Guzman et al. supplementary material

## Supplementary material

To view supplementary material for this article, please visit https://doi.org/10.1017/S1463423624000136
