# Supplementary material for: General practitioner preferences for telehealth consultations in Australia: a pilot survey and discrete choice experiment
Source: Prim Health Care Res Dev. 2024 May 9;25:e28. doi: 10.1017/S1463423624000136 (PMC11091543; doi:10.1017/S1463423624000136)
Supplement: De Guzman et al. supplementary material [file S1463423624000136sup001.docx]

# Supplementary files

## Supplementary file 1: General telehealth questions and direct GP preferences

| Characteristic  (n=60) | | Mean (SD) |
| --- | --- | --- |
| Telehealth knowledge/experience (1 being none and 10 being knowledgeable/experienced) | | 7.3 (1.5) |
| Length of time (months) spent delivering videoconference consultations | | 5.6 (7.9) |
|  |  | **N (%)** |
| Effect of bulk billing restrictions on GP business | Good effect | 39 (65.0) |
|  | None or limited effect | 9 (15.0) |
|  | Bad effect | 12 (20.0) |
| Resources required for videoconference consultation compared to in-person consultations (n=27) | | |
| Technology resources | Less | 5 (18.5) |
|  | Same | 15 (55.6) |
|  | More | 7 (25.9) |
| Technology support | Less | 2 (7.4) |
|  | Same | 8 (29.6) |
|  | More | 17 (63.0) |
| Administration or support staff time | Less | 3 (11.1) |
|  | Same | 14 (51.9) |
|  | More | 10 (37.0) |
| Longer patient consult times | Less | 9 (27.0) |
|  | Same | 11 (40.7) |
|  | More | 7 (25.9) |
| Consultation space or location | Less | 4 (14.8) |
|  | Same | 19 (70.4) |
|  | More | 4 (14.8) |
| Resources required for telephone consultation compared to in-person consultations  (n=60) | | |
| Technology resources | Less | 22 (36.7) |
|  | Same | 34 (56.7) |
|  | More | 4 (6.7) |
| Technology support | Less | 22 (36.7) |
|  | Same | 28 (46.7) |
|  | More | 10 (16.7) |
| Administration or support staff time | Less | 18 (30.0) |
|  | Same | 24 (40.0) |
|  | More | 18 (30.0) |
| Longer patient consult times | Less | 31 (51.7) |
|  | Same | 20 (33.3) |
|  | More | 9 (15.0) |
| Consultation space or location | Less | 22 (36.7) |
|  | Same | 30 (50.0) |
|  | More | 8 (13.3) |
| Preferences for consultation mode for different consultation lengths, all participants (n=60) | |  |
| Preferred mode for 5-minute consultations | No preference | 10 (16.7) |
|  | In-person consultation | 12 (20.0) |
|  | Telephone consultation | 32 (53.3) |
|  | Videoconference consultation | 6 (10.0) |
| Preferred mode for 40-minute consultations | No preference | 4 (6.7) |
|  | In-person consultation | 26 (43.3) |
|  | Telephone consultation | 10 (16.7) |
|  | Videoconference consultation | 20 (33.3) |
| Preferences for consultation mode for different consultation lengths, telephone experience only (n=33) | |  |
| Preferred mode for 5-minute consultations | No preference | 6 (18.2) |
|  | In-person consultation | 8 (24.2) |
|  | Telephone consultation | 13 (39.4) |
|  | Videoconference consultation | 6 (18.2) |
| Preferred mode for 40-minute consultations | No preference | 4 (12.1) |
|  | In-person consultation | 10 (30.3) |
|  | Telephone consultation | 10 (30.3) |
|  | Videoconference consultation | 9 (27.3) |
| Preferences for consultation mode for different consultation lengths, telephone and videoconference experience (n=27) | |  |
| Preferred mode for 5-minute consultations | No preference | 4 (14.8) |
|  | In-person consultation | 4 (14.8) |
|  | Telephone consultation | 19 (70.4) |
|  | Videoconference consultation | 0 (0.0) |
| Preferred mode for 40-minute consultations | No preference | 0 (0.0) |
|  | In-person consultation | 16 (59.3) |
|  | Telephone consultation | 0 (0) |
|  | Videoconference consultation | 11 (40.7) |
| Preferences for consultation mode for different patient presentations, telephone experience only (n=33) | | |
| Follow-up | In-person | 72.7 |
|  | Videoconference | 6.1 |
|  | Telephone | 21.2 |
| Repeat prescription | In-person | 36.4 |
|  | Videoconference | 39.4 |
|  | Telephone | 24.2 |
| Test results | In-person | 36.4 |
|  | Videoconference | 21.2 |
|  | Telephone | 42.4 |
| Hypertension | In-person | 66.7 |
|  | Videoconference | 9.1 |
|  | Telephone | 24.2 |
| Lipid Disorder | In-person | 39.4 |
|  | Videoconference | 27.3 |
|  | Telephone | 33.3 |
| Diabetes | In-person | 33.3 |
|  | Videoconference | 42.4 |
|  | Telephone | 24.2 |
| Depression | In-person | 66.7 |
|  | Videoconference | 9.1 |
|  | Telephone | 24.2 |
| Anxiety | In-person | 45.5 |
|  | Videoconference | 27.3 |
|  | Telephone | 27.3 |
| URTI | In-person | 27.3 |
|  | Videoconference | 42.4 |
|  | Telephone | 30.3 |
| Asthma | In-person | 63.6 |
|  | Videoconference | 9.1 |
|  | Telephone | 27.3 |
| Acute bronchitis | In-person | 45.5 |
|  | Videoconference | 30.3 |
|  | Telephone | 24.2 |
| Arthritis | In-person | 33.3 |
|  | Videoconference | 27.3 |
|  | Telephone | 39.4 |
| Back complaint | In-person | 48.5 |
|  | Videoconference | 27.3 |
|  | Telephone | 24.2 |
| GORD | In-person | 42.4 |
|  | Videoconference | 21.2 |
|  | Telephone | 36.4 |
| UTI | In-person | 42.4 |
|  | Videoconference | 21.2 |
|  | Telephone | 36.4 |
| Pain | In-person | 39.4 |
|  | Videoconference | 24.2 |
|  | Telephone | 36.4 |
| Preferences for consultation mode for different patient presentations, telephone and videoconference experience (n=27) | | |
| Follow-up | In-person | 29.6 |
|  | Videoconference | 11.1 |
|  | Telephone | 59.3 |
| Repeat prescription | In-person | 22.2 |
|  | Videoconference | 14.8 |
|  | Telephone | 63.0 |
| Test results | In-person | 37.0 |
|  | Videoconference | 22.2 |
|  | Telephone | 40.7 |
| Hypertension | In-person | 59.3 |
|  | Videoconference | 11.1 |
|  | Telephone | 29.6 |
| Lipid Disorder | In-person | 40.7 |
|  | Videoconference | 22.2 |
|  | Telephone | 37.0 |
| Diabetes | In-person | 66.7 |
|  | Videoconference | 14.8 |
|  | Telephone | 18.5 |
| Depression | In-person | 55.6 |
|  | Videoconference | 18.5 |
|  | Telephone | 25.9 |
| Anxiety | In-person | 48.1 |
|  | Videoconference | 22.2 |
|  | Telephone | 29.6 |
| URTI | In-person | 40.7 |
|  | Videoconference | 18.5 |
|  | Telephone | 40.7 |
| Asthma | In-person | 70.4 |
|  | Videoconference | 11.1 |
|  | Telephone | 18.5 |
| Acute bronchitis | In-person | 44.4 |
|  | Videoconference | 18.5 |
|  | Telephone | 37.0 |
| Arthritis | In-person | 51.9 |
|  | Videoconference | 25.9 |
|  | Telephone | 22.2 |
| Back complaint | In-person | 66.7 |
|  | Videoconference | 11.1 |
|  | Telephone | 22.2 |
| GORD | In-person | 40.7 |
|  | Videoconference | 14.8 |
|  | Telephone | 44.4 |
| UTI | In-person | 59.3 |
|  | Videoconference | 11.1 |
|  | Telephone | 29.6 |
| Pain | In-person | 59.3 |
|  | Videoconference | 7.4 |
|  | Telephone | 33.3 |
